# Correlation between hemoglobin-to-albumin ratio and complications after radical gastrectomy in gastric cancer patients

**DOI:** 10.3389/fmed.2025.1683276

**Published:** 2025-10-23

**Authors:** Wenbin Luo, Ruoyun Li, Chaofan Pan, Changjiang Luo

**Affiliations:** ^1^The Second Clinical Medical School, Lanzhou University, Lanzhou, China; ^2^Department of General Surgery, The Second Hospital of Lanzhou University, Lanzhou, China

**Keywords:** hemoglobin-to-albumin ratio, radical gastrectomy, gastric cancer, postoperative complications, Clavien-Dindo classification, nutritional biomarker

## Abstract

**Background:**

Patients who undergo radical gastrectomy for gastric cancer often experience multiple complications that affect the recovery process and reduce quality of life. The hemoglobin-to-albumin ratio (HAR) reflects nutritional status and is widely used to assess various diseases. However, its association with complications after radical gastrectomy in gastric cancer patients has not been fully explored.

**Methods:**

This study retrospectively analyzed clinical and pathological data from 352 gastric cancer patients who underwent radical gastrectomy at the Second Hospital of Lanzhou University between January 2023 and December 2024. The severity of postoperative complications was graded using the Clavien-Dindo classification system, resulting in two groups: non-complication group (*n* = 254) and Clavien-Dindo ≥II group (*n* = 98). Multivariate logistic regression models were employed to assess the correlation between HAR and Clavien-Dindo ≥II grade complications. Subsequently, sensitivity analysis, subgroup analysis, restricted cubic spline (RCS) modeling, and threshold effect evaluation were conducted.

**Results:**

Multivariate logistic regression analysis showed that for every 1-unit increase in HAR, the risk of Clavien-Dindo ≥II grade complications decreased by 62.0% (95% CI: 0.188–0.749; *p* < 0.05). Sensitivity analysis confirmed this conclusion, demonstrating that patients with higher HAR levels exhibited a 64.4% lower risk of complications compared to patients with lower HAR levels (95% CI: 0.170–0.726; *p* < 0.05). Restricted cubic spline (RCS) analysis demonstrated a negative correlation between HAR and complications, and the threshold effect analysis determined the critical point of HAR to be 2.87. Subgroup analysis showed that most subgroups did not exhibit significant differences in interaction *p* values. Interestingly, a significant interaction was observed between HAR and platelet (interaction *p* = 0.011).

**Conclusion:**

This study indicates that higher HAR is significantly and negatively correlated with the risk of complications. Higher levels of HAR effectively prevent Clavien-Dindo ≥ II-grade complications. Consequently, HAR can serve as a clinically useful indicator for predicting complications following radical gastrectomy for gastric cancer.

## 1 Introduction

Gastric cancer constitutes a substantial component of the global cancer burden, with age-standardized incidence and mortality rates ranking fifth and fourth, respectively, among all malignant tumors (1). Radical surgery is the standard treatment for resectable gastric cancer (2). Despite its notable efficacy in enhancing the five-year survival rate of patients (3), postoperative complications persist as a pivotal factor influencing patient recovery and quality of life. According to the Clavien-Dindo postoperative complication grading system (4, 5), the incidence of grade II or higher complications requiring drug treatment or invasive intervention in patients undergoing radical gastrectomy for gastric cancer can reach 25% (6). These complications are significantly associated with prolonged hospital stays, increased medical costs, and reduced three-year survival rates (7).

Current research indicates that preoperative nutritional support can optimize surgical outcomes through multiple mechanisms (8). First, it can regulate excessive inflammatory responses and reduce levels of pro-inflammatory factors such as tumor necrosis factor-*α* (TNF-α) and interleukin-6 (IL-6). Second, studies have confirmed that it can enhance cellular immune function, manifested by increased lymphocyte activity and antibody production capacity (9). The dual effect has been demonstrated to have the capacity to markedly diminish the likelihood of postoperative infectious complications and systemic complications. Patients with malnutrition may experience increased resting energy expenditure, anorexia, loss of adipose tissue, muscle wasting, and reduced protein synthesis, which can lead to severe postoperative complications classified as Clavien-Dindo grade III or higher (10). In the context of clinical nutritional assessment, hemoglobin and albumin serve as pivotal biomarkers, reflecting the body’s oxygen transport capacity and protein reserves. Preoperative hypoalbuminemia has been demonstrated to exhibit an independent association with an augmented risk of postoperative pulmonary infection (11). Concurrent testing of these biomarkers is of significant clinical value. Extensive evidence-based medical evidence indicates that preoperative hypoalbuminemia and low hemoglobin levels are significantly associated with shorter postoperative survival and increased complication rates in patients with malignant tumors (12).

In recent years, the hemoglobin-to-albumin ratio (HAR) has garnered significant attention as a novel composite indicator that integrates nutritional status with inflammatory conditions (13). Hemoglobin, the oxygen-carrying protein, directly reflects tissue oxygen supply and the severity of anemia (14). Albumin plays a central role in maintaining plasma colloid osmotic pressure and acts as a negative acute-phase protein involved in systemic inflammatory regulation (15). The HAR has the capacity to simultaneously capture dual information regarding oxygen-carrying capacity and inflammatory/nutritional status, thus offering a more comprehensive assessment value compared to single indicators. Unlike traditional nutritional indices such as the prognostic nutritional index (PNI), platelet-to-albumin ratio (PAR), and controlling nutritional status (CONUT) score, which primarily focus on nutritional or inflammatory components alone, HAR provides a more integrative perspective that better reflects the complex physiological interplay in surgical patients. Clinical studies has demonstrated that HAR is associated with the prognosis of various diseases, including colorectal cancer and nasopharyngeal cancer (16, 17). However, there is a paucity of research investigating the correlation between HAR and postoperative complications in patients undergoing radical gastrectomy for gastric cancer.

The study aimed to assess the predictive value of HAR in postoperative complications by exploring the correlation between postoperative complications and HAR following radical gastrectomy. The goal was to provide new reference indicators for the perioperative management of gastric cancer patients in clinical practice, enabling the early identification and timely intervention for high-risk patients, reducing the risk of postoperative complications, and improving patient outcomes and quality of life.

## 2 Materials and methods

### 2.1 Data and participants

This retrospective study examined 397 gastric cancer patients who underwent radical gastrectomy at the Second Hospital of Lanzhou University between January 2023 and December 2024. These patients underwent radical proximal, distal, or total gastrectomy based on tumor location, size, and stage. Complete tumor resection was confirmed through intraoperative macroscopic observation and postoperative pathology, which demonstrated the absence of residual tumor.

This study was conducted in accordance with the Declaration of Helsinki and approved by the Medical Ethics Committee of the Second Hospital of Lanzhou University (Project Number:2025 A-652). As this study is a retrospective and the privacy and personal identity information of the patients were protected, the need for informed consent was waived by the Medical Ethics Committee of the Second Hospital of Lanzhou University.

### 2.2 Inclusion and exclusion criteria

**Figure 1:**
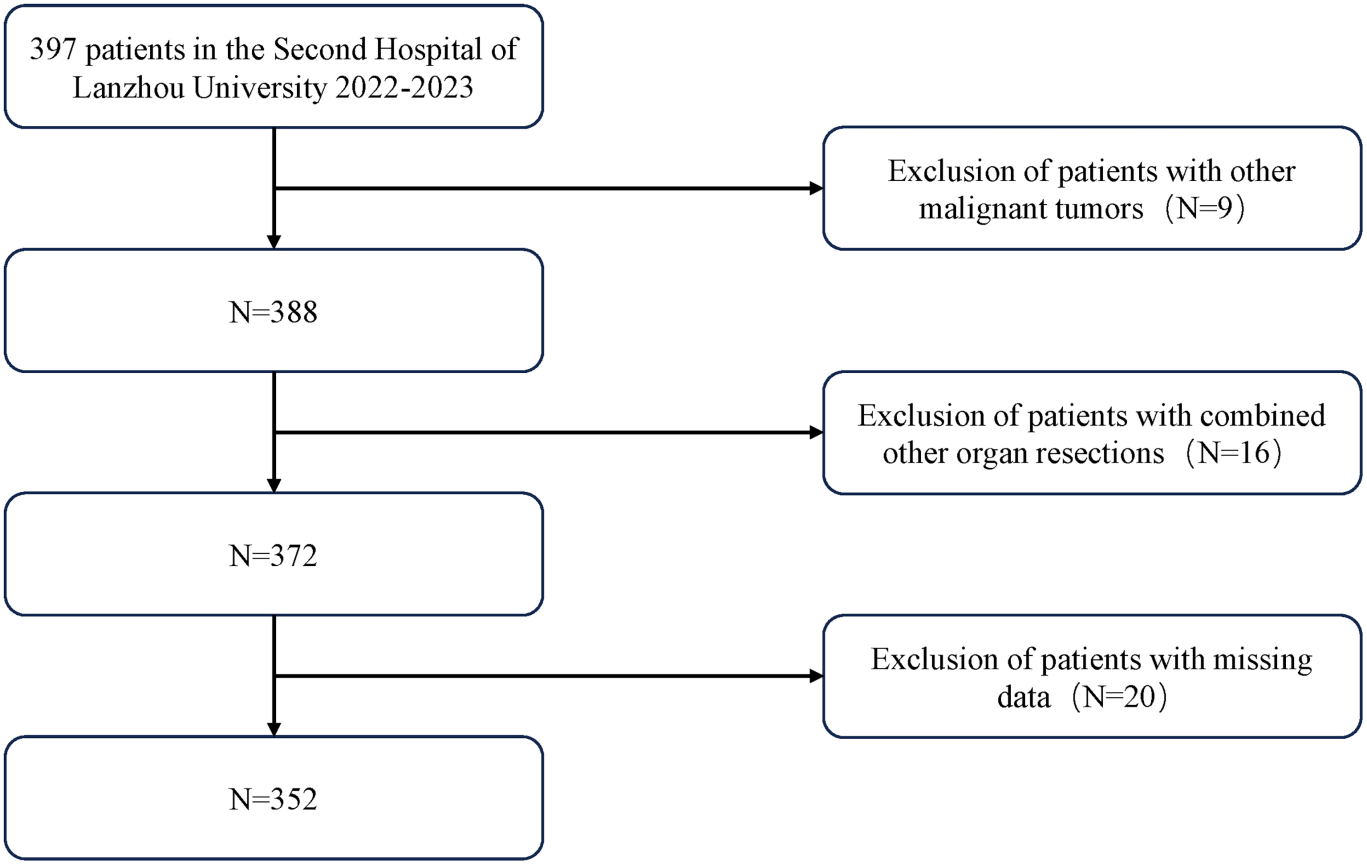
Flow diagram of the selection of eligible patients.

Inclusion criteria are as follows: (1) Pathologically confirmed gastric cancer post-surgery, patients underwent radical resection of the proximal, distal, or entire stomach; (2) No evidence of tumor invasion into adjacent organs or distant metastasis; (3) Data required for outcome measures are complete and comprehensive. Exclusion criteria include: (1) Patients with gastroesophageal junction cancer who underwent open thoracotomy; (2) Patients who underwent gastric stump surgery, recurrent cancer surgery, or combined surgery with other organ surgeries; (3) Patients with special gastric tumors, including lymphoma, hepatoma, neuroendocrine tumors, or gastrointestinal stromal tumors (GISTs). The final sample included 352 patients (Figure 1).

### 2.3 Assessment of Clavien-Dindo ≥II grade complications

Postoperative complications are issues that arise from surgery within 30 days after the procedure. The diagnosis of these conditions is made through the observation of clinical symptoms, the execution of laboratory tests, and the analysis of imaging examinations. These findings are then meticulously documented in the patient’s medical records. These complications include pulmonary infection, intra-abdominal infection, wound infection, anastomotic obstruction, pleural effusion, intra-abdominal hemorrhage, anastomotic leakage or fistula, acute heart failure, acute respiratory failure, and multiple organ dysfunction, among others (Supplementary Table S1).

The severity of complications is classified according to the Clavien-Dindo classification system, which is structured into five grades. Level one is characterized by mild symptoms that do not pose a threat to life, while level five complications are associated with fatality (Supplementary Table S2). Patients with Clavien-Dindo ≥ II are defined as the complication group, while the remaining patients are defined as the non-complication group.

### 2.4 Clinical variables

The case data to be observed and collected in this study include: medical history, postoperative recovery status, serological indicators, and risk factors associated with postoperative complications. Medical history includes the presence of hypertension, diabetes, cardiovascular and cerebrovascular diseases, smoking history, alcohol consumption history, neoadjuvant chemotherapy, and history of abdominal surgery. Variables related to postoperative recovery include the status of postoperative recovery, whether complications occurred, and the treatment measures taken for complications. For patients with multiple complications, grading is based on the severity of the most severe complication. Serological tests are conducted for all admitted cases. Relevant serological data were collected, including white blood cell count (WBC), neutrophil count (NEU), lymphocyte count (LYM), monocyte count (MONO), hemoglobin (HGB), red blood cell distribution width standard deviation (RDWSD), red blood cell distribution width coefficient of variation (RDWCV), platelet count (PLT), albumin (ALB), total cholesterol count (TC), lactate dehydrogenase (LDH), and blood fibrinogen (FIB). Additionally, collect and assess potential risk factors associated with postoperative complications, including sex, age, surgical method, TNM stage, intraoperative blood loss, and operation time. HAR was calculated as the hemoglobin-to-albumin ratio using the most recent routine laboratory results within 24–48 h before anesthesia. To avoid reverse causality, pre-intervention values were used if patients received transfusion, albumin, parenteral nutrition, or large-volume fluids during this period.

### 2.5 Statistical analysis

This study employed various statistical methods for a systematic analysis of the data. Descriptive statistical analysis was first performed on demographic characteristics. Continuous variables were expressed as mean ± SD and compared between groups using T-tests or Kruskal-Wallis rank sum tests. Categorical variables were presented as percentages and compared using Chi-square tests. For further optimization of variable selection, Lasso regression was employed. Lasso regression, with L1 regularization, shrinks the coefficients of less important variables to zero, achieving dimensionality reduction and enhancing the model’s generalizability, thereby preventing overfitting. To ensure model stability and minimize the risk of multicollinearity, the Variance Inflation Factor (VIF) was used to screen independent variables. VIF quantifies the level of collinearity among variables, and variables with a VIF greater than 5 were excluded to improve model stability and interpretability.

Furthermore, to capture potential non-linear relationships between HAR and Clavien-Dindo ≥ II-grade complications, Restricted Cubic Spline (RCS) analysis was applied. Multivariable logistic regression was used to calculate odds ratios (ORs) and 95% confidence intervals (CIs) to assess the association between HAR and Clavien-Dindo ≥ II-grade complications. The optimal HAR cutoff value was determined through threshold effect analysis. Additionally, sensitivity analysis was performed by excluding outliers below the 1st percentile and above the 99th percentile in HAR. Subgroup analysis was also conducted to further explore the relationship between HAR and Clavien-Dindo ≥ II-grade complications.

All statistical analyses were performed using R software (version 4.4.2).

## 3 Results

### 3.1 Baseline characteristics of participants

The present study comprised 352 patients diagnosed with gastric cancer who underwent radical gastrectomy. The subjects were divided into two groups: a complication group and a non-complication group. This division was based on the presence or absence of Clavien-Dindo ≥ II-grade complications postoperatively. As demonstrated in Table 1, the preliminary demographic characteristics analysis indicated that the mean age of the complication group (65.2 ± 8.5 years) was considerably higher than that of the non-complication group (57.7 ± 9.4, *p* < 0.001). With respect to hemoglobin (HGB) levels, the complication group (119.5 ± 27.3) exhibited significantly lower levels in comparison to the non-complication group (139.0 ± 26.6, *p* < 0.001). The operation time in the complication group (3.8 ± 0.9 h) was significantly longer than that in the non-complication group (4.5 ± 1.0, *p* < 0.001). Furthermore, the complication group exhibited significantly elevated levels of white blood cell count (WBC), neutrophil count (NEU), monocyte count (MONO), and platelet count (PLT) (*p* < 0.001). There were no significant differences in gender distribution between the two groups; however, the proportion of elderly patients in the complication group (75.5%) was significantly higher than that in the non-complication group (39.0%, *p* < 0.001). The prevalence of diabetes mellitus in the complication group (17.3%) was significantly higher than that in the non-complication group (5.1%, *p* < 0.001). However, there were no significant differences in the prevalence of cardiovascular disease between the two groups. Furthermore, the HAR index in the complication group was 3.5, which is significantly higher than the 3.2 in the non-complication group.

**Table 1:** Population characteristics analysis of patients from 2023 to 2024.

Characteristic	Non-complication	Complication	*P*-value
*N*	254	98	
Age (year)	57.7 ± 9.4	65.2 ± 8.5	<0.001
WBC (10^3^/μL)	5.4 ± 1.6	6.7 ± 2.1	<0.001
NEU (10^3^/μL)	3.3 ± 1.3	4.6 ± 1.9	<0.001
LYM (10^3^/μL)	1.6 ± 0.6	1.4 ± 0.6	0.005
MONO (10^3^/μL)	0.4 ± 0.1	0.5 ± 0.2	<0.001
HGB (g/L)	139.0 ± 26.6	119.5 ± 27.3	<0.001
RDWSD (fL)	47.8 ± 7.8	50.8 ± 10.0	0.005
RDWCV (%)	14.2 ± 2.8	15.9 ± 3.9	<0.001
PLT (10^3^/μL)	215.4 ± 73.5	264.7 ± 96.3	<0.001
ALB (g/L)	39.2 ± 4.0	37.4 ± 4.3	<0.001
TC (mmol/L)	4.0 ± 0.9	3.7 ± 0.9	<0.001
LDH (U/L)	184.4 ± 58.7	199.3 ± 68.9	0.011
FIB (g/L)	2.9 ± 0.6	3.5 ± 0.8	<0.001
CEA (μg/L)	6.7 ± 21.7	8.1 ± 23.1	0.117
HAR	3.5 ± 0.6	3.2 ± 0.6	<0.001
Diabetes, *n* (%)			<0.001
No	241 (94.9%)	81 (82.7%)	
Yes	13 (5.1%)	17 (17.3%)	
Hypertension, *n* (%)			0.048
No	219 (86.2%)	76 (77.6%)	
Yes	35 (13.8%)	22 (22.4%)	
Heartdisease, *n* (%)			0.3622
No	246 (96.9%)	93 (94.9%)	
Yes	8 (3.1%)	5 (5.1%)	
Smoking, *n* (%)			0.991
No	215 (84.6%)	83 (84.7%)	
Yes	39 (15.4%)	15 (15.3%)	
Drinking, *n* (%)			0.112
No	246 (96.9%)	98 (100.0%)	
Yes	8 (3.1%)	0 (0.0%)	
Abdominal surgery, *n* (%)			0.26
No	224 (88.2%)	82 (83.7%)	
Yes	30 (11.8%)	16 (16.3%)	
Sex, *n* (%)			0.984
Male	205 (80.7%)	79 (80.6%)	
Female	49 (19.3%)	19 (19.4%)	
Surgical method, *n* (%)			0.128
Proximal	17 (6.7%)	12 (12.2%)	
Distal	127 (50.0%)	40 (40.8%)	
Total	110 (43.3%)	46 (46.9%)	
T			0.221
1	51 (20.1%)	13 (13.3%)	
2	42 (16.5%)	13 (13.3%)	
3	85 (33.5%)	33 (33.7%)	
4	76 (29.9%)	39 (39.8%)	
N			0.379
0	101 (39.8%)	34 (34.7%)	
1	48 (18.9%)	14 (14.3%)	
2	42 (16.5%)	18 (18.4%)	
3	63 (24.8%)	32 (32.7%)	
TNM			0.028
1	76 (29.9%)	16 (16.3%)	
2	72 (28.3%)	30 (30.6%)	
3	106 (41.7%)	52 (53.1%)	
Intraoperative blood loss (mL)	89.6 ± 76.1	86.8 ± 87.1	0.699
Operation time (h)	3.8 ± 0.9	4.5 ± 1.0	<0.001

WBC, white blood cell count; NEU, neutrophil count; LYM,: lymphocyte count; MONO, monocyte count; HGB, hemoglobin.

RDWSD, red blood cell distribution width standard deviation; RDWCV, red blood cell distribution width coefficient of variation; PLT, platelet count; ALB, albumin; TC, total cholesterol count; LDH, lactate dehydrogenase; FIB, plasma fibrinogen; CEA, carcinoembryonic antigen; HAR, hemoglobin-to-albumin ratio.

### 3.2 Lasso regression and variance inflation factor analysis

**Figure 2:**
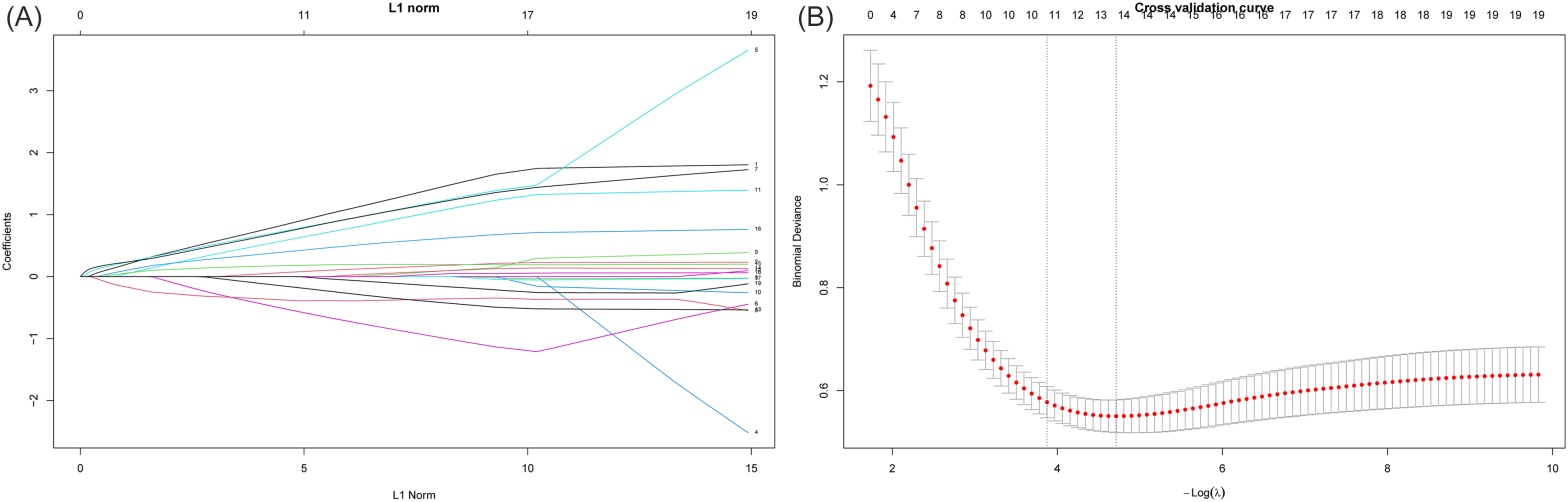
Screening of variables based on Lasso regression. This figure illustrates the process of optimizing variable selection through Lasso regression. In 5-fold cross validation, alpha = 1 is used for L1 regularization, and the optimal regularization parameter *λ* = 0.008985436 is determined by minimizing the cross validation error. This parameter optimized the performance of the model, and through the *λ* value, Lasso regression screened out 13 significant variables. **(A)** The characteristics of the regression coefficients of each variable as a function of L1 regularization intensity. **(B)** The process of selecting the optimal *λ* value through cross validation in the Lasso regression model.

To further optimize variable selection, we selected 18 significant variables from Table 1 for Lasso regression analysis, including age, white blood cell count (WBC), neutrophil count (NEU), lymphocyte count (LYM), monocyte count (MONO), hemoglobin (HGB), red blood cell distribution width standard deviation (RDWSD), red blood cell distribution width coefficient of variation (RDWCV), platelet count (PLT), albumin (ALB), total cholesterol count (TC), lactate dehydrogenase (LDH), plasma fibrinogen (FIB), HAR index, diabetes status (Diabetes), hypertension status (Hypertension), TNM stage, and Operation time. We set alpha = 1 for standard L1 regularization. Lasso regression achieves feature selection and dimension reduction by penalizing model coefficients, causing the coefficients of unimportant variables to approach zero. This method helps reduce model complexity and prevent overfitting. During model optimization, the optimal *λ* value (*λ* min = 0.008985436) was determined using 5-fold cross-validation, and the model was retrained using this *λ* value, ultimately incorporating 13 variables (as shown in **Figure** **2**).

Subsequently, we used VIF analysis to address potential multicollinearity issues among variables. The results of the variance inflation factor analysis (Table 2) showed that after excluding hemoglobin level (HGB) and red blood cell distribution width standard deviation (RDWSD), the variance inflation factor values of all remaining variables were less than 5, indicating that there were no multicollinearity issues.

**Table 2:** Variance inflation factor.

Variables	VIF1	VIF2
Age	1.702	1.689
Diabetes	1.088	1.083
NEU	1.630	1.580
LYM	1.832	1.811
MONO	1.933	1.926
HGB	5.537	NA
RDWSD	1.712	1.497
PLT	2.029	2.012
TC	1.251	1.192
LDH	1.203	1.155
FIB	1.157	1.142
Operation time	1.218	1.213
HAR	4.481	1.543

NEU, neutrophil count; LYM, lymphocyte count; MOMO, monocyte count; HGB, hemoglobin; RDWSD, red blood cell distribution width standard deviation; PLT, platelet count; TC, total cholesterol count; LDH, lactate dehydrogenase; FIB, plasma fibrinogen; HAR, hemoglobin-to-albumin ratio.

### 3.3 Association between HAR and Clavien-Dindo ≥II grade complications

Following a two-way stepwise regression analysis, the final model was determined, encompassing seven significant factors: age, neutrophil count (NEU), lymphocyte count (LYM), monocyte count (MONO), platelet count (PLT), total cholesterol count (TC), operation time. To further investigate the relationship between HAR and Clavien-Dindo ≥II grade complications, three multivariable regression models were assessed (Table 3). In Model 1, which did not adjust for covariates, each 1-unit increase in HAR was associated with a 61.6% lower risk of complications (OR = 0.384, 95% CI: 0.255–0.568, *p* < 0.001). In Model 2, which adjusted for covariates including age, neutrophil count (NEU), lymphocyte count (LYM), the odds ratio (OR) for HAR and complications was 0.274 (95% CI: 0.158–0.459, *p* < 0.001). In Model 3, which included all covariates, the OR for HAR was further adjusted to 0.380 (95% CI: 0.188–0.749, *p* = 0.006). Furthermore, with increasing HAR, the risk of complications continued to decrease significantly (P for trend <0.005).

**Table 3:** Multivariable logistic regression models for the association between HAR and Clavien-Dindo ≥II grade complications.

Variables	Crude Model1^a^		Model2^b^		Model3^c^	
	OR (95%CI)	*P* value	OR (95%CI)	*P* value	OR (95%CI)	*P* value
HAR	0.384(0.255–0.568)	<0.001	0.274(0.158–0.459)	<0.001	0.380(0.188–0.749)	0.006
Categories						
Q1	Reference		Reference		Reference	
Q2	0.556 (0.298–1.028)	0.063	0.278 (0.120–0.618)	0.002	0.646 (0.208–1.965)	0.443
Q3	0.346(0.176–0.662)	0.002	0.193(0.079–0.448)	<0.001	0.445(0.148–1.310)	0.144
Q4	0.218(0.102–0.439)	<0.001	0.114(0.043–0.283)	<0.001	0.172(0.047–0.579)	0.006
*P* for trend	<0.001		<0.001		0.006	

HAR, hemoglobin-to-albumin ratio.

^a^Without adjustment.

^b^Adjusted for age, NEU, LYM.

^c^Adjusted for all variables.

^d^*P* for trend is calculated by converting the quartiles of HAR into level variables, assigning values of 0, 1, 2, and 3, and then inputting the level variables into the regression model.

**Figure 3:**
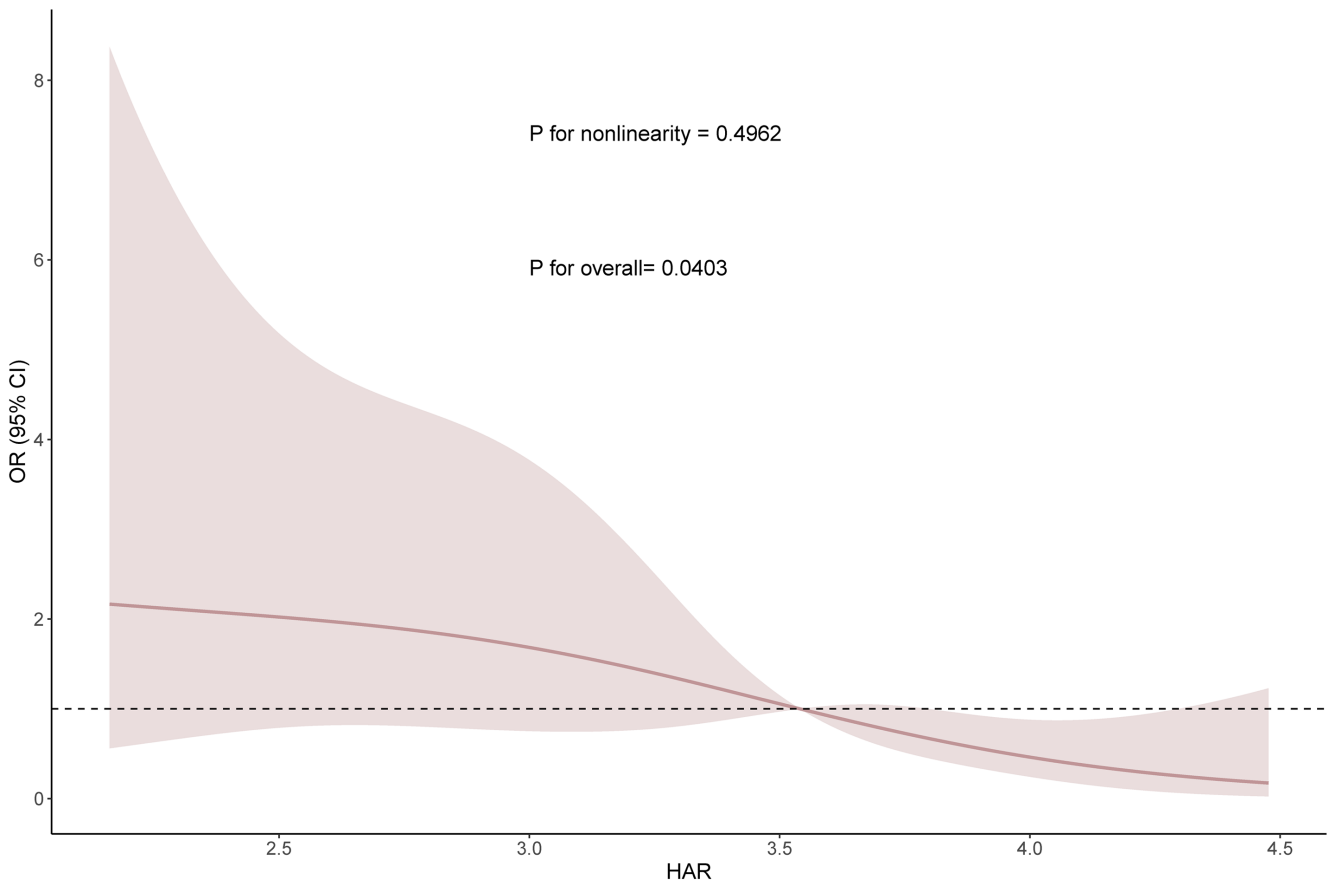
Smooth curve fitting (RCS analysis) between HAR and complications based on Model 3.

Based on Model 3, we performed restricted cubic spline analysis to further explore the relationship between HAR and Clavien-Dindo ≥II complications (Figure 3). The results showed that there was no nonlinear relationship between HAR and complications (nonlinear test *p* value = 0.4962). To further elucidate the nature of this relationship, we subsequently conducted a threshold effect analysis (Table 4). Threshold effect analysis showed that no significant association was observed when the HAR value was below 2.87; however, when the HAR value was equal to or above 2.87, there was a significant negative correlation with event risk (OR, 0.21; 95% CI: 0.06–0.76; *p* = 0.018).

**Table 4:** Threshold effect analysis of HAR and complications using a two-piecewise logistic regression model.

Threshold effect analysis	OR (95%CI)	*P*
Inflection point	2.87	
<2.87	1.32 (0.05–37.08)	0.871
≥2.87	0.21 (0.06–0.76)	0.018
*P* for likelihood test		0.408

### 3.4 Sensitivity analysis

Sensitivity analysis showed that after excluding the upper and lower 1% of outliers before and after HAR (Table 5), the association remained statistically significant, with an adjusted odds ratio (OR) of 0.175 (95% confidence interval, 0.048–0.593; *p* = 0.006), indicating a significant negative correlation between the highest HAR group and event risk. Trend analysis further supported the conclusion that higher HAR values are associated with lower event risk (trend *p*-value = 0.006). The ROC curve shows that the AUC of HAR is higher than that of PNI, PAR, and CONUT score (Supplementary Figure S1).

**Table 5:** Sensitivity analysis.

Variables	OR	CI_lower	CI_upper	*P* for trend
HAR	0.356	0.170	0.726	0.005
Categories				
Q1	Reference		Reference	Reference
Q2	0.613	0.196	1.870	0.392
Q3	0.436	0.145	1.280	0.133
Q4	0.175	0.048	0.593	0.006
*P* for trend	0.006			

*P* for trend is calculated by converting the quartiles of HAR into level variables, assigning values of 0, 1, 2, and 3, and then inputting the level variables into the regression model.

### 3.5 Subgroup analyses

**Figure 4:**
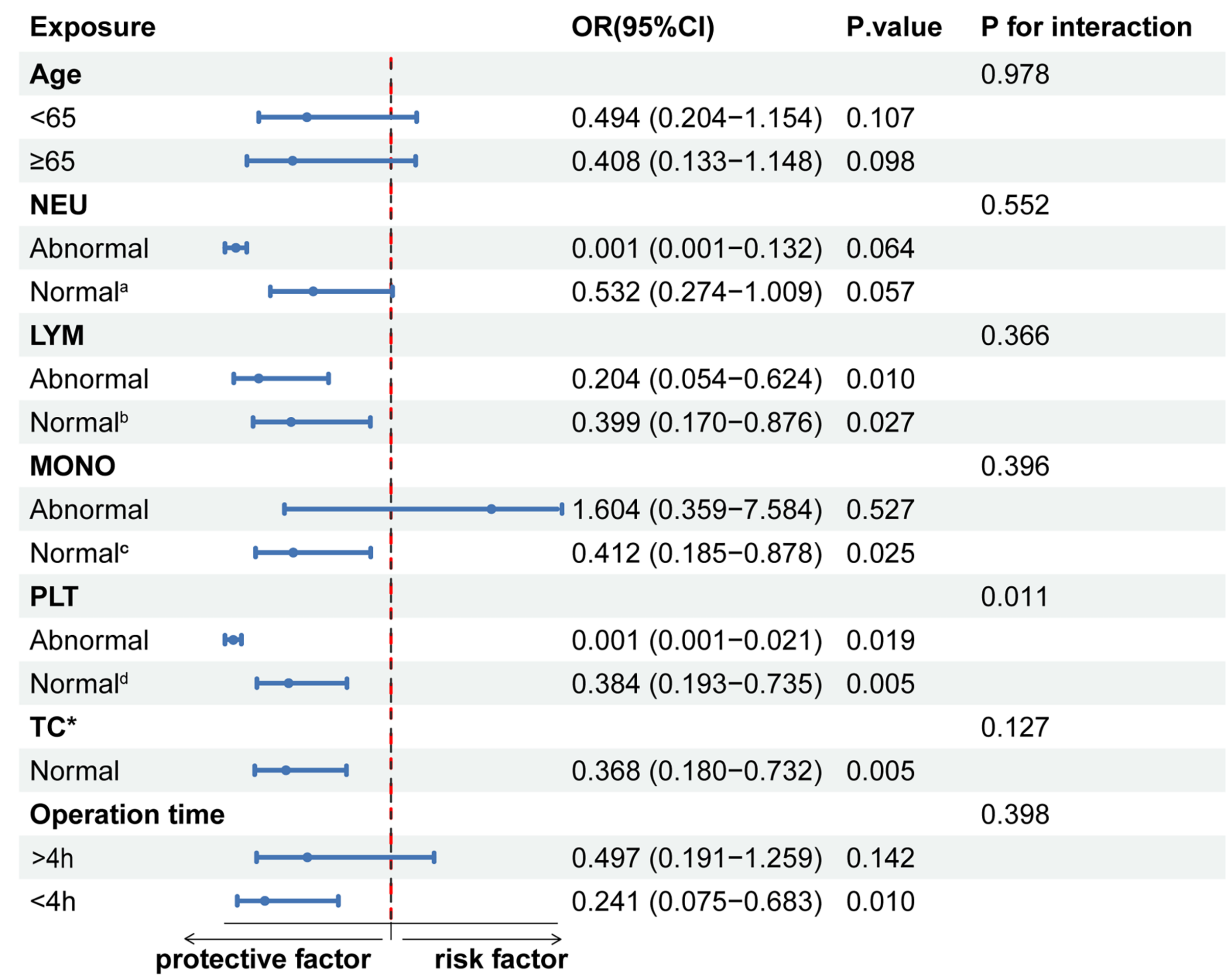
Subgroup stratified analysis of HAR and Clavien-Dindo ≥II grade complications. a: 1.8–6.3 × 10^9^/L; b: 1.1–3.2 × 10^9^/L; c: 0.1–0.6 × 10^9^/L; d: 125–350 × 10^9^/L. *Insufficient data for the abnormal total cholesterol (TC) group.

Further subgroup analyses (Figure 4) showed that HAR was generally negatively associated with the risk of Clavien-Dindo ≥II grade complications. This trend was observed in both the normal lymphocyte (LYM) group (OR, 0.399, 95% CI, 0.170–0.876), the abnormal group (OR, 0.204, 95% CI, 0.054–0.624), monocytes (MONO) normal group (OR, 0.412, 95% CI, 0.185–0.878), and total cholesterol (TC) normal group (OR, 0.368, 95% CI, 0.180–0.732), and also in patients with operation time <4 h(OR, 0.241, 95% CI, 0.075–0.683). Interestingly, HAR had a protective effect against complications in both the normal platelet (PLT) group (OR, 0.384, 95% CI, 0.193–0.735) and the abnormal platelet group (OR, 0.001, 95% CI, 0.001–0.021), and there was a significant interaction between the two groups (*P* for interaction = 0.011).

## 4 Discussion

This study retrospectively analyzed clinical data from 352 gastric cancer patients who underwent radical gastrectomy at the Second Hospital of Lanzhou University between January 2023 and December 2024. The findings indicated a substantial negative correlation between hemoglobin-to-albumin ratio (HAR) and Clavien-Dindo ≥II grade complications. Specifically, after adjusting for all covariates in Model 3, each 1-unit increase in HAR was associated with a 62.0% reduction in the risk of postoperative complications. Furthermore, the occurrence of complications was found to be significantly lower in the high HAR group compared to the low HAR group, exhibiting a statistically significant overall trend (*P* for trend = 0.006). RCS analysis further revealed a linear negative correlation between HAR and Clavien-Dindo ≥II grade complications. A subsequent threshold effect analysis identified a significant negative correlation between HAR values of 2.87 or higher and the risk of postoperative complications. Sensitivity analysis further confirmed the stability and consistency of the study results. Also, HAR outperformed established indices such as PNI, PAR, and CONUT in predicting postoperative complications.

HAR is a novel nutritional biomarker calculated based on albumin and hemoglobin. Previous studies have shown that low preoperative HAR is associated with short survival in gastric cancer patients (18). HAR’s clinical significance primarily lies in its integration of hemoglobin and albumin, two fundamental physiological indicators. These indicators influence postoperative complications in gastric cancer patients through a complex network of interactions that regulate oxygen transport, inflammatory balance, and immune homeostasis (19). Compared with established indices such as PNI, PAR, and CONUT, which mainly capture nutritional or inflammatory status alone, HAR provides a more comprehensive reflection of both oxygen-carrying capacity and systemic inflammation, thereby offering superior prognostic value.

Gastric cancer commonly reduces hemoglobin through multiple, reinforcing mechanisms. Friable tumor mucosa and abnormal neovasculature lead to chronic occult or overt bleeding (20). Tumor-associated inflammation elevates hepcidin levels, sequesters iron, shortens red cell lifespan, and diminishes erythropoietin responsiveness, resulting in anemia of inflammation (21). Moreover, reduced dietary intake, gastric outlet obstruction, and mucosal atrophy or malabsorption contribute to deficiencies in iron, folate, and vitamin B12 (10). This sustained, disease-specific downward pressure on hemoglobin decreases oxygen-carrying capacity, lowers tissue oxygen tension, and activates the HIF-1α signaling pathway (22). The ensuing hypoxic response upregulates VEGF expression and promotes the formation of hypoxia-driven but functionally inadequate neovessels, which compromises microvascular perfusion, delays tissue repair, and increases the risk of postoperative complications such as anastomotic leaks (23). In parallel, gastric cancer often lowers albumin through a tumor-driven negative acute-phase response, cancer-related malnutrition, and cachexia (24). Concurrently, low albumin levels decrease plasma colloid osmotic pressure, causing tissue edema and accelerating the spread of inflammatory factors (25). This impairs albumin’s antioxidant function, leading to elevated pro-inflammatory factor levels and triggering an inflammatory cascade reaction. This increases the risk of sepsis (26, 27). Furthermore, reduced hemoglobin and albumin levels can impair immune cell function by causing mitochondrial hypoxia in T cells, decreasing natural killer (NK) cell activity, impairing lymphocyte proliferation, and activating the complement system (28), thereby increasing the incidence of postoperative infection and abdominal abscesses (29, 30). Threshold effect analysis shows that the HAR index has no statistically significant effect on postoperative complications when it is less than 2.87. At this point, patients often have severe anemia and hypoalbuminemia, which trigger complications dominated by confounding factors, such as multiple organ failure and systemic inflammation (31). These factors mask the effect of HAR. However, when the HAR index is ≥2.87, a 1-unit increase in the HAR index reduces the risk of postoperative complications by 82.0%. In summary, the HAR index significantly influences the occurrence of postoperative complications in gastric cancer patients and can serve as an important biomarker for risk assessment.

Subgroup analysis revealed that the protective effect of HAR was observed across various subgroups, including those defined by age, neutrophil count, lymphocyte count, monocyte count, and total cholesterol level. HAR significantly reduced the risk of Clavien-Dindo ≥II-grade complications through its dual mechanisms of optimizing oxygen transport via hemoglobin and attenuating the inflammatory cascade via albumin. Notably, this protective effect was observed in both the normal and abnormal platelet count groups, although it appeared stronger in patients with abnormal platelet levels. Mechanistically, abnormal platelet counts reflect a state of metabolic and inflammatory stress, which is frequently associated with coagulation disorders, platelet hyperreactivity, and microcirculatory dysfunction (32). These alterations aggravate tissue hypoxia and trigger amplification of inflammatory cascades. Under such conditions, higher hemoglobin levels may compensate by enhancing oxygen-carrying capacity, thereby alleviating hypoxic stress at the tissue level (33). Hemoglobin also promotes VEGF-mediated angiogenesis under hypoxic conditions, which facilitates microvascular repair and oxygen redistribution (34). Albumin, on the other hand, counteracts the deleterious consequences of platelet dysfunction and inflammation. It exerts antioxidant effects by scavenging reactive oxygen species, reduces endothelial injury, and downregulates NF-κB signaling, thereby attenuating inflammation-driven vascular permeability (35, 36). In addition, albumin stabilizes the endothelial glycocalyx, which is otherwise disrupted by platelet-derived inflammatory mediators, and preserves microvascular barrier integrity (37). Together, these mechanisms provide resilience against the detrimental effects of platelet abnormalities, explaining the stronger protective role of HAR in the abnormal platelet group. Even in patients with normal platelet counts, perioperative trauma and surgical stress invariably activate systemic inflammation and coagulation pathways (38). In this context, a higher HAR still offers protection by maintaining physiological balance, reflecting the integrated contribution of oxygen delivery, nutritional reserves, and inflammatory modulation.

### 4.1 Limitations

This study has several limitations. First, although HAR consistently showed a negative association with postoperative complications across multivariable regression, sensitivity, and RCS analyses, a potential concern is that both hemoglobin and albumin may increase during nutritional recovery, raising the question of whether their ratio could fluctuate unpredictably. However, these biomarkers reflect distinct physiological domains that often change at different rates. Thus, HAR should not be regarded as a pure nutritional marker but rather as a composite index integrating anemia, protein reserves, and systemic inflammation. Nevertheless, in extreme cases of severe anemia combined with hypoalbuminemia, its predictive value may be limited, and future studies should consider combining HAR with established nutritional scoring systems for validation. Second, this was a single-center retrospective study, which may restrict the generalizability of the findings. Third, although we adjusted for multiple established risk factors, some potential confounders were not fully captured, such as detailed perioperative nutritional interventions (e.g., enteral nutrition) and certain comorbidities (e.g., chronic liver disease). Fourth, current evidence supports validation of HAR solely in gastric cancer. Its applicability to other malignancies remains uncertain and should be assessed in prospective, disease-specific multicenter cohorts with external validation before clinical extrapolation. Finally, the retrospective design inherently carries the risk of selection and information bias. Therefore, our findings should be interpreted with caution, and prospective multicenter studies with more comprehensive data collection are warranted to further confirm and extend these results.

## 5 Conclusion

This study found a significant negative linear correlation between higher HAR levels and the risk of Clavien-Dindo grade II or higher complications in gastric cancer patients. Higher HAR levels were associated with a lower risk of postoperative complications. Specifically, for every one-unit increase in HAR, the risk of complications decreases by 62.0%. This relationship is statistically significant when HAR values are ≥2.87. These results support the potential of HAR as a predictive tool for postoperative Clavien-Dindo ≥II-grade complications in gastric cancer patients, providing an important foundation for early diagnosis and targeted interventions in clinical practice.

## Data Availability

The raw data supporting the conclusions of this article will be made available by the authors, without undue reservation.
