# Supplementary material for: Correlation between hemoglobin-to-albumin ratio and complications after radical gastrectomy in gastric cancer patients
Source: Front Med (Lausanne). 2025 Oct 23;12:1683276. doi: 10.3389/fmed.2025.1683276 (PMC12588993; doi:10.3389/fmed.2025.1683276)
Supplement: Supplementary file 1 [file Table_1.docx]

| Table S1 The 98 complication cases following radical gastrectomy and their complication grading | | | | | | |
| --- | --- | --- | --- | --- | --- | --- |
| Postoperative complications | **Clavien-Dindo classifications (2009 version)** | | | | | |
|  | Ⅱ | Ⅲa | Ⅲb | Ⅳa | Ⅳb | Ⅴ |
| Intra-abdominal hemorrhage | 28 | 1 | 0 | 0 | 0 | 0 |
| Pulmonary infection | 9 | 0 | 0 | 0 | 0 | 0 |
| Intra-abdominal infection | 10 | 0 | 0 | 0 | 0 | 0 |
| Pleural effusion | 0 | 4 | 0 | 0 | 0 | 0 |
| Acute respiratory failure | 0 | 0 | 0 | 1 | 1 | 0 |
| Phlebothrombosis | 7 | 0 | 0 | 0 | 0 | 0 |
| Wound infection | 4 | 0 | 1 | 0 | 0 | 0 |
| Anastomotic obstruction | 4 | 0 | 0 | 0 | 0 | 0 |
| Anastomotic leakage or fistula | 2 | 0 | 0 | 0 | 0 | 0 |
| Acute heart failure | 6 | 0 | 0 | 1 | 0 | 0 |
| Abdominal fluid | 8 | 10 | 0 | 0 | 0 | 0 |
| Cerebral infarction | 1 | 0 | 0 | 0 | 0 | 0 |

| Table S2 Classification of Surgical Complications | |
| --- | --- |
| Grades | Definition |
| Grade I | Any deviation from the normal postoperative recovery process requires anti-nausea, anti-vomiting, antipyretic, analgesic, and diuretic drugs; electrolyte supplementation; and physical therapy. |
| Grade II | Requiring pharmacological treatment with drugs other than such allowed for grade I complications. Blood transfusions and total parenteral nutrition are also included. |
| Grade III | Requiring surgical, endoscopic or radiological intervention |
| III-a | intervention not under general anesthesia |
| III-b | intervention under general anesthesia |
| Grade IV | Life-threatening complication, requiring ICU-management |
| IV-a | single organ dysfunction (including dialysis) |
| IV-b | multi organ dysfunction |
| Grade V | Death |

*ICU: Intensive care unit*


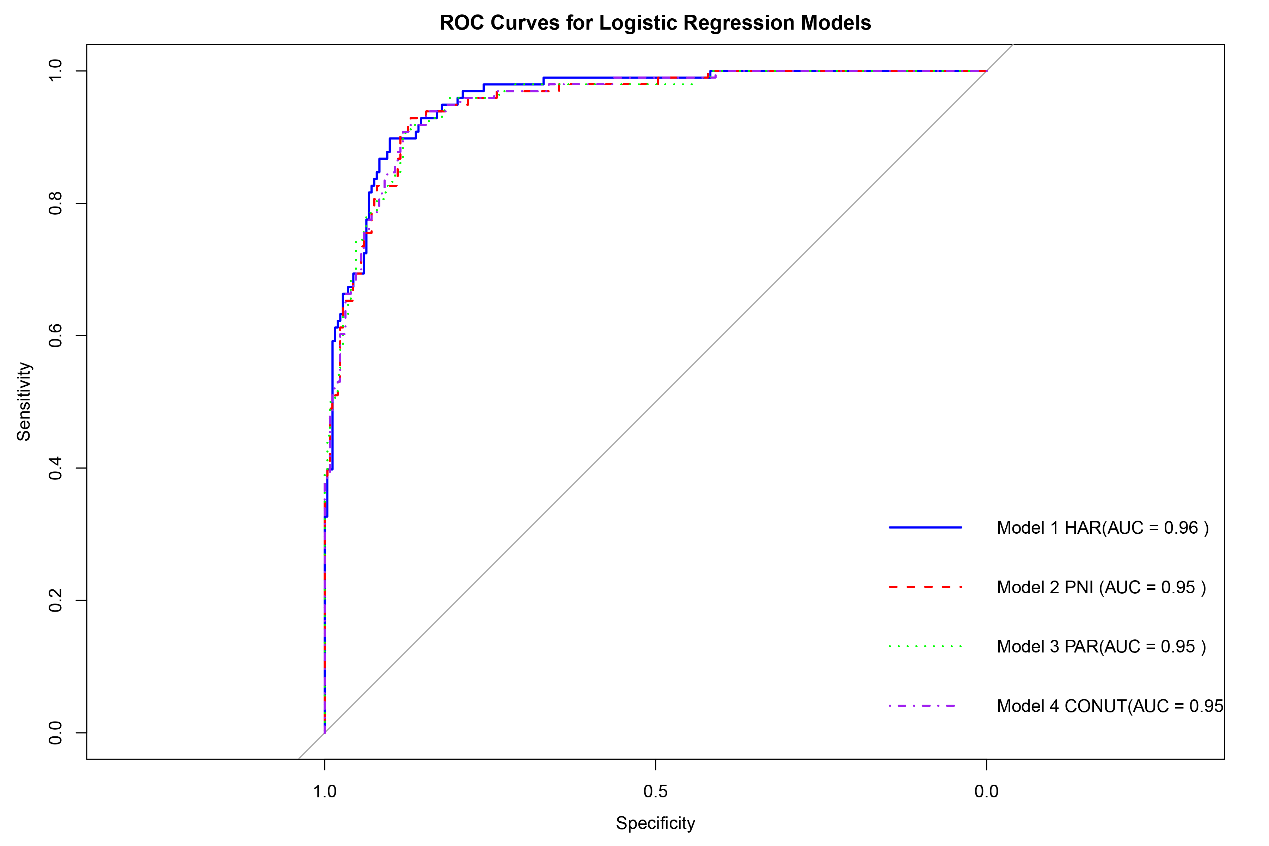


**Figure S1** ROC curve
