# Supplementary figures and images for: Correlation between hemoglobin-to-albumin ratio and complications after radical gastrectomy in gastric cancer patients
Source: Front Med (Lausanne). 2025 Oct 23;12:1683276. doi: 10.3389/fmed.2025.1683276 (PMC12588993; doi:10.3389/fmed.2025.1683276)

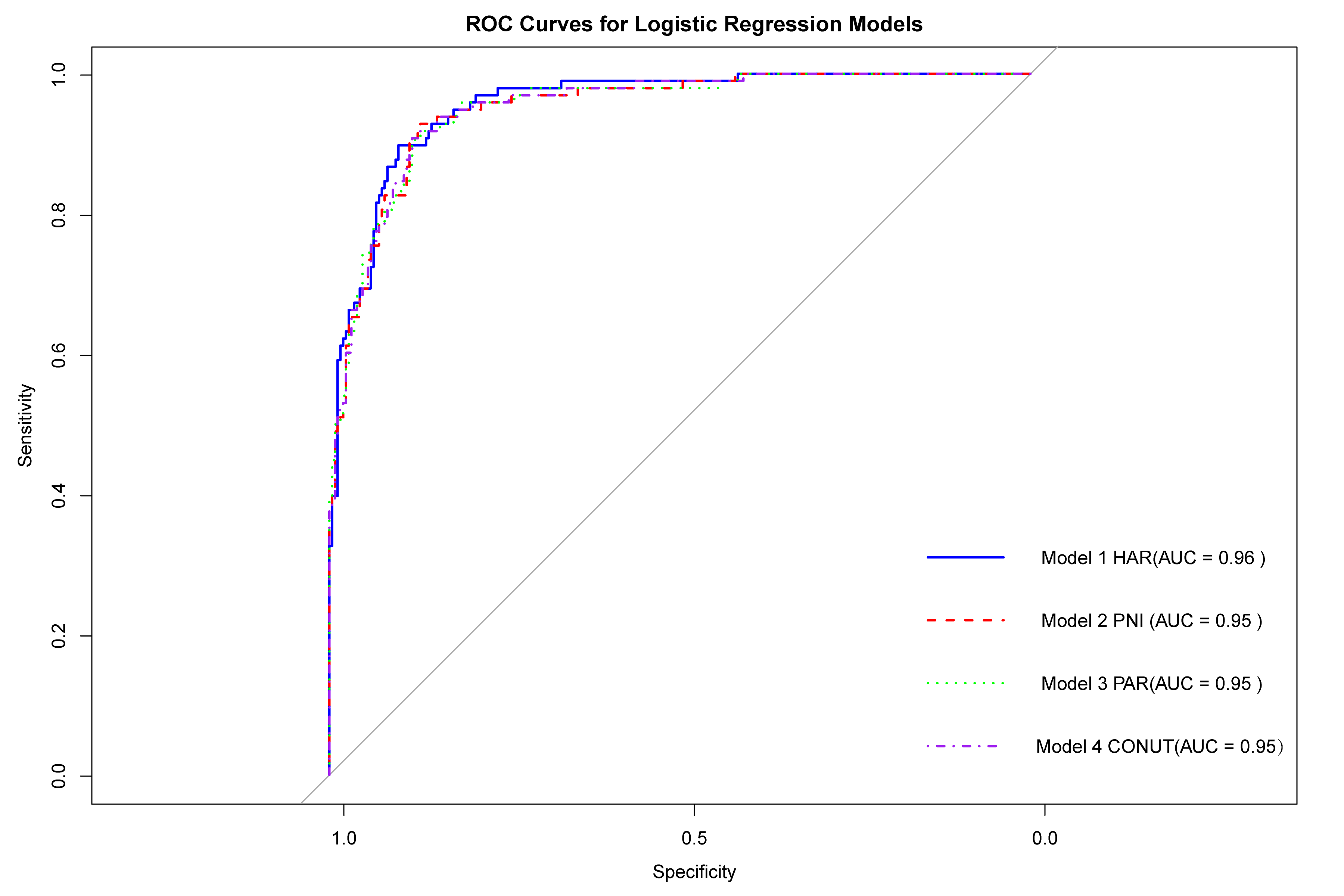

Supplement: Supplementary file 2 [file Image_1.tif]
